# A Systematic Review on the Effects of *Epichloë* Fungal Endophytes on Drought Tolerance in Cool-Season Grasses

**DOI:** 10.3389/fpls.2021.644731

**Published:** 2021-03-24

**Authors:** Facundo A. Decunta, Luis I. Pérez, Dariusz P. Malinowski, Marco A. Molina-Montenegro, Pedro E. Gundel

**Affiliations:** ^1^Universidad de Buenos Aires, Consejo Nacional de Investigaciones Científicas y Técnicas, Instituto de Investigaciones Fisiológicas y Ecológicas Vinculadas a la Agricultura (IFEVA), Facultad de Agronomía, Buenos Aires, Argentina; ^2^Texas A&M AgriLife Research, Vernon, TX, United States; ^3^Instituto de Ciencias Biológicas, Universidad de Talca, Talca, Chile; ^4^Centro de Estudios Avanzados en Zonas Áridas (CEAZA), Universidad Católica del Norte, Coquimbo, Chile; ^5^Centro de Investigaciones y Estudios Avanzados del Maule (CIEAM), Universidad Católica del Maule, Talca, Chile

**Keywords:** symbiosis, mutualism, abiotic stress, wild grasses, domesticated grasses, water shortage, meta-analysis

## Abstract

Symptomless fungal endophytes in the genus *Epichloë* are repeatedly mentioned to increase tolerance of cool-season grasses to a wide range of environmental stress factors, mainly drought. However, the generality of this idea is challenged because (i) most studies have been conducted on two economically important forage grasses {tall fescue [*Festuca arundinacea* (Schreb.) Dumort] and perennial ryegrass (*Lolium perenne* L.)}, (ii) endophyte-mediated mechanisms and effects on plant responses to drought have shown to be highly variable across species, and that (iii) symbiosis incidence in plant populations occurring in extremely arid environments is usually low. We question this idea by reviewing the existing information about *Epichloë* fungal endophyte effects on drought tolerance in cool-season grasses. We combined standard review, vote counting, and calculation of effect sizes to synthesize the literature, identify information gaps, and guide future research. The total number of studies was higher for domesticated than for wild species, a ratio that was balanced when papers with data quality for effect size calculus were considered. After the drought, endophyte-infected plants accumulated more aboveground and belowground biomass than non-infected counterparts, while no effect on tillering was observed. However, these effects remained significant for wild (even on tillering) but not for domesticated species. Interestingly, despite the continuous effort in determining physiological mechanisms behind the endophyte effects, no studies evaluated plant fecundity as a measure of ecological fitness nor vital rates (such as survival) as to escalate individual-level variables to population. Together with the high variability in results, our work shows that generalizing a positive effect of fungal endophytes in plant tolerance to drought may be misleading. Future studies combining field surveys with manipulative experiments would allow us to unravel the role of fungal endophytes in plant adaptation by considering the evolutionary history of species and populations to the different ecological contexts.

## Introduction

An increasing interest exists in understanding the role of symbiotic microorganisms on plant phenotypic adjustment to environmental stresses (Redman et al., 2011; Shankar Naik, 2019; Acuña-Rodríguez et al., 2020). It is suggested that under different scenarios of global climate change, microbial symbionts will be fundamental for plants to face novel stressful conditions (Redman et al., 2011; Dastogeer, 2018). Drought is one of the most worrisome aspects of climate change, as its increase in frequency and severity will directly affect the primary productivity of natural and managed ecosystems (Dai, 2011; IPCC, 2018; Volaire, 2018; Slette et al., 2019). Here, we performed a critical review and analysis of the existing experimental evidence for the established idea that *Epichloë* fungal endophytes improve the ability of host grasses to withstand drought events.

Certain grasses of the tribe Poeae (subfamily: Poöideae) establish symbiosis with fungal endophytes of genus *Epichloë* (family: Clavicipitaceae; phylum: Ascomycota; order Hypocreales) (Schardl, 2010). Although the symbiosis discovery dates from the early twentieth century (Freeman, 1904), it became important in 1977 and 1981 when domestic animals' intoxications in the United States and New Zealand were associated with fungal endophytes colonizing aerial tissues of tall fescue (*Festuca arundinacea*, syn. *Schedonorus arundinaceus, Lolium arundinaceum*) and perennial ryegrass (*Lolium perenne*) (Bacon et al., 1977; Fletcher and Harvey, 1981). It is well-known today that fescue toxicosis and ryegrass staggers are animal diseases caused by alkaloids (ergovaline and lolitrem-B) produced by their common associated fungi *Epichloë coenophiala* and *Epichloë festucae*, respectively (Schardl et al., 2013; Leuchtmann et al., 2014). The importance of these two forage grass species in temperate regions of the world triggered an enormous research activity aimed at understanding the endophyte effects not only on animal health and growth but also on plant performance, stand persistence, and forage production (Bacon, 1993; Malinowski and Belesky, 2000, 2019; Klotz, 2015). Removing fungal endophytes from grass cultivars was an alternative to avoid the antiquality traits (i.e., toxic alkaloids) in forage; however, this was also associated with a poor performance of grass plants in the field (Bouton et al., 2001). Another strategy was the reinfection of elite grass cultivars with selected (“friendly”) endophytes, which do not produce toxic alkaloids but still produce others such as lolines and peramine that protect host plants against pests (Bouton et al., 2002; Gundel et al., 2013; Johnson et al., 2013; Lugtenberg et al., 2016).

Including the above-mentioned grasses, there are so far more than 50 other species that have been described to host *Epichloë* fungal endophytes covering a vast range of ecosystems all over the world (Semmartin et al., 2015). Motivated by its conspicuous characteristics (namely, “the vertical transmission” and “the alkaloid-mediated defense of hosts against herbivores”), the grass–endophyte symbiosis became a model system of studies in ecology and evolution (Saikkonen et al., 2006; Rudgers et al., 2009; Gundel et al., 2011; Omacini et al., 2012; Hume et al., 2020). In fact, the studies of factors driving plant–endophyte specificity and environmental controls favoring or not the symbiosis incidence in populations have been among the major goals in research (Malinowski and Belesky, 2006, 2019; Schardl et al., 2008; Rudgers et al., 2009; Karimi et al., 2012; Iannone et al., 2013; Semmartin et al., 2015; Schirrmann et al., 2018). However, it has been not straightforward to explain the driving environmental factors behind the distribution and abundance of symbiotic grasses in nature (Saikkonen et al., 2006; Rudgers et al., 2009; Gundel et al., 2011, 2016; Semmartin et al., 2015; Wang et al., 2020). Besides, the identification of a strong bias in the published literature toward studies conducted with domesticated grasses (tall fescue and the ryegrasses) led to the conclusion that they were unable to fully represent the diversity of interaction outcomes of the grass–endophyte symbiosis in natural ecosystems (Saikkonen et al., 2006).

The endophyte-conferred resistance to herbivory has been well-documented with fungal-produced alkaloids as responsible secondary metabolites to the impaired performance of herbivores (Clay, 1988; Saikkonen et al., 2013; Schardl et al., 2013; Bastias et al., 2017). However, underlying mechanisms to the endophyte-mediated improvement of plant tolerance to abiotic stress factors—such as drought—have been inconsistent (Malinowski and Belesky, 2000; Hamilton et al., 2012; Dastogeer, 2018; Wang et al., 2020). The idea that fungal endophytes improve plant tolerance to drought triggered ecological hypotheses and field surveys predicting high endophytic incidence in populations toward arid extremes of natural precipitation gradients (Lewis et al., 1997; Gibert et al., 2012; Afkhami et al., 2014). However, there has been evidence contradicting such predictions: in extremely arid settings, the incidence of endophytes is low (see Novas et al., 2007; Gundel et al., 2011; Semmartin et al., 2015). Motivated by this observation, we have recently pointed out that inferring the causal mechanisms of *Epichloë* fungal endophyte effects on host plant performance from short-term and controlled drought experiments with domesticated grass species may be misleading to understand the abundance and distribution of the grass–endophyte symbiosis in nature (Gundel et al., 2016).

Here, we question the widely accepted idea that *Epichloë* fungal endophytes confer host grass tolerance to drought, as it is not enough supported by experimental evidence to generalize across plant and fungus species and environmental conditions. Besides being the first step for a quantitative review, an analysis of the scientific literature is particularly important for synthesizing information, uncover biases, identify knowledge gaps, and guide future research (see e.g., Saikkonen et al., 2006; Nunez-Mir et al., 2016; Gurevitch et al., 2018; Sayer, 2018). We conducted a critical synthesis, summarizing the existing information about *Epichloë* fungal endophyte effects on drought tolerance in grasses. We specifically addressed the presumption that there would be a bias of information toward *F. arundinacea* first and followed by *L. perenne*, due to their importance as domesticated forage grasses. Then, we predicted that the effect of endophytes for those domesticated species will substantially differ from that observed in wild grasses. Although our ultimate goal was to carry out a quantitative assessment, a big proportion of the published studies (especially for *F. arundinacea*) failed to meet the requirements for being included in the meta-analysis (see section Materials and Methods). Therefore, we summarized the published information on the topic by complementing the calculation of effect sizes with standard review and vote counting.

## Materials and Methods

The literature review consisted of searching for papers meeting the criteria of having “endophyte” AND (“*Acremonium*” OR “*Epichloë*” OR “*Neotyphodium*”) AND “drought” in their title, abstract, or keywords. The searching was conducted in September 2020, with no lower date limit, and performed with the scientific search engine Scopus (www.scopus.com). We included the genus *Acremonium* because, in some early articles, it was used for designating *Epichloë*/*Neotyphodium* endophytes. We extended our search by analyzing the references within each paper. In total, we found 190 research articles published between 1983 and 2020. After a careful check on the list, we discarded all those working with true *Acremonium* species. Non-experimental articles were excluded (review articles, commentary, or perspective papers). The whole process of selection is shown in a Preferred Reporting Items for Systematic Reviews and Meta-Analyses (PRISMA) flow diagram in Supplementary Figure 1.

We filtered the articles that fit into the following criteria: (i) endophyte-infected and non-infected plants were subjected to a drought treatment; (ii) at least one measure of plant performance was reported (e.g., biomass, number of tillers, survival, or any physiological variable); and (iii) the data included means, a measure of variation (standard deviation or standard error), and three or more independent replicates for each treatment. After that, the database consisted of 87 cases (experiments) from 26 studies (papers) (Supplementary Figure 1). For each case, we also extracted species names of both the host plant and the endophyte fungus (Supplementary Table 1). Grass species were classified into domesticated or wild species following the paper by Glémin and Bataillon (2009). Given that many studies did not meet criteria iii, we used vote counting as a summary of the existing information. Although we are aware of the limitations of such a simple alternative (Koricheva and Gurevitch, 2013; Lortie, 2014), it can be still useful to visualize the existing information on a given topic put all together.

We included multiple observations per study when the data reported came from independent experiments or different plant populations. Nonetheless, when different measures were reported from the same experiment, we selected the last response variable/s on plants after the drought condition was stopped. Additionally, in cases where other treatments were included (e.g., different nutrient levels), we selected data from the control condition to avoid factors' confounding effects. For quantitative analysis, the mean, the standard deviation, and the sample size were extracted from the article (criteria iii). When presented in figures, data were extracted using the GetData software (getdata-graph-digitizer.com). For each case, we calculated an effect size using the standardized mean difference metric between endophyte-infected and non-infected plants, and its confidence interval in the “metafor” package 1.9-8 version in r 3.2.3 (Viechtbauer, 2010). The effect size was considered significant if the confidence interval did not overlap with zero (Koricheva and Gurevitch, 2013). A positive effect size means that endophyte infection improved plant performance under drought stress. We also estimated the level of consistency among studies by calculating between-studies heterogeneity (τ_2_ and associated Q statistics; Viechtbauer, 2010). Because τ_2_ is dependent on sample size, we calculated I_2_-values, providing a standardized estimate of total heterogeneity ranging from 0 to 1. We used several approaches to verify that our results were not impacted by publication bias (Koricheva and Gurevitch, 2013), namely, an inspection of funnel plots and calculation of fail-safe numbers.

## Results

### Search and Data Collection

**Figure 1:**
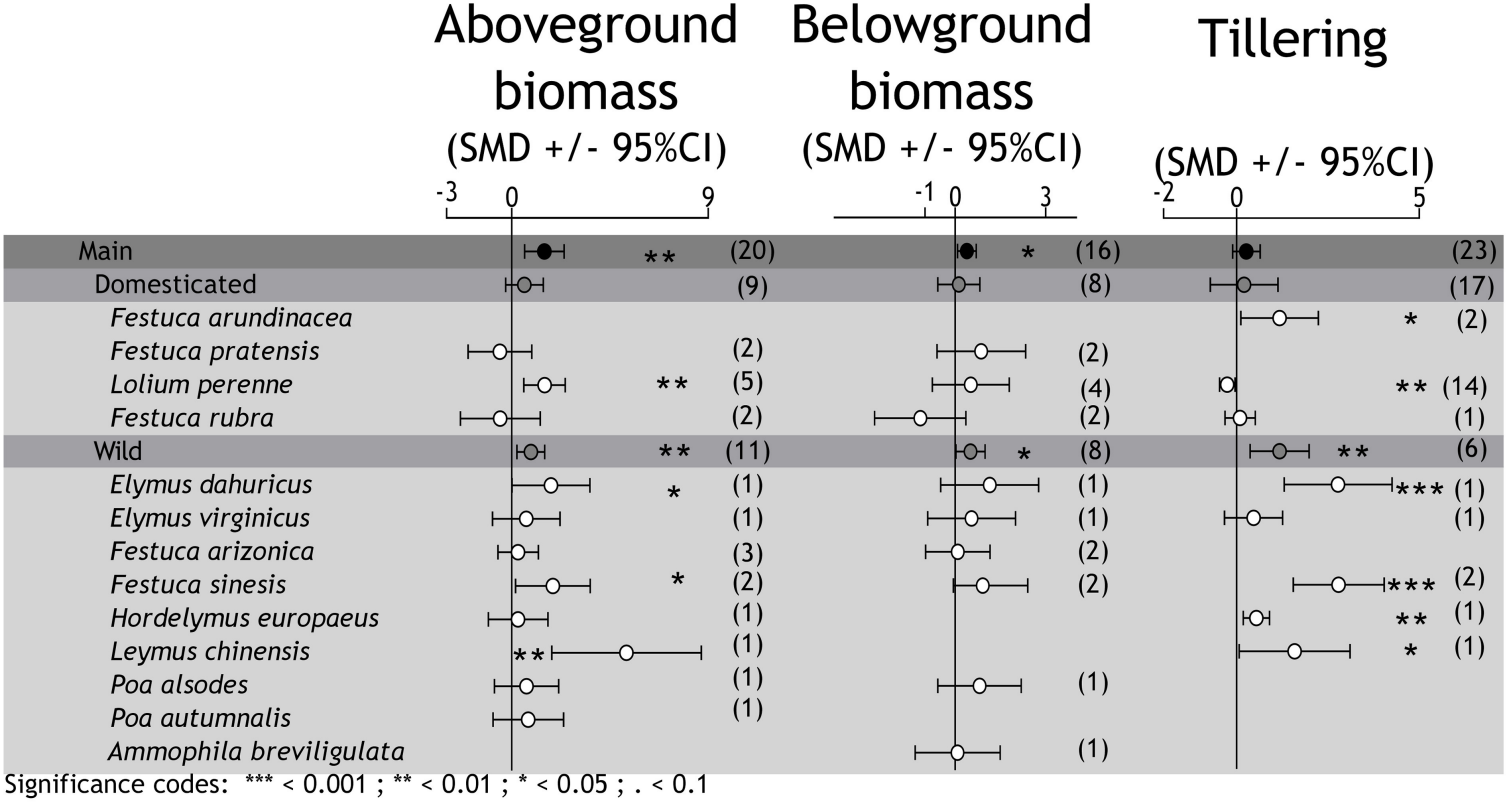
The effects of *Epichloë* fungal endophytes on grass species performance (aboveground biomass, belowground biomass, and the number of tillers) under drought conditions. Mean effect sizes (SMD, standardized mean difference) and confidence intervals (95%CI) for the comparisons between E+ and E– plants. The symbiotic associations are grouped in domesticated and wild grass species. The number of studies is indicated on the right to each value between parentheses.

Our search recovered a total of 190 publications, of which 83 were specific about the topic addressed here, that is, the endophyte-mediated responses of plants to drought. From the above-mentioned studies, only 26 were experimental, while the remaining 57 were reviews, conference, or perspective papers, or had no factorial designs for testing the differential performance of endophyte-infected and non-infected plants under drought. Those latter studies were field surveys of endophyte incidence along aridity gradients or in environments with contrasting precipitation regimes. The word “drought” in those non-specific but experimental articles (*n* = 107) was present in the abstract and therefore caught by the searching (Figure 1; Supplementary Material). However, since water availability was not experimentally manipulated in those works, they were not included in the calculations.

Of the 83 articles that were about endophyte effect on host plant responses to drought, not all were appropriate for a quantitative approach (i.e., meta-analysis) mainly because most of them did not report either the number of replicates or a variation measure around mean values (Supplementary Figure 1). Due to that, we made a summary table with vote counting as a descriptive synthesis of the literature on the topic. *F. arundinacea* and *L. perenne* were the most studied species with 16 (≈30%) and 13 (≈24%) experiments each, respectively. Apart from those species and except for *Achnatherum inebrians, Festuca arizonica, Festuca pratensis*, and *Festuca rubra*, which have on average three studies each, all the other 11 species have been studied once (Table 1). Most studies used biomass [either total (aboveground + belowground) or just aboveground] and the number of tillers as integrative response variables of plant performance (Table 1). For both response variables, the number of studies yielding positive, neutral, or negative results of the endophytes on plant performance under drought was evenly distributed across species. No studies were found evaluating the impact of drought on plant fitness (i.e., fecundity or seed production).

**Table 1:** Summary table showing the number of experiments describing beneficial (+), neutral (0), or detrimental (–) effect of an *Epichloë* fungal endophyte on the host grass performance variables (biomass, tiller, and survival), under drought conditions.

**Host Species**	**Biomass**					**Tillering**					**Survival**				
	**+**	**0**	**–**	**NE**	**Net effect**	**+**	**0**	**–**	**NE**	**Net effect**	**+**	**0**	**–**	**NE**	**Net effect**
*Achnatherum inebrians*	2			1	2	1			2	1				3	NE
*Agrostis hyemalis*			1		−1				1	NE				1	NE
*Ammophila breviiigulata*	2				2				1	NE				1	NE
*Bromus laevipes*		1			0				1	NE	1				1
*Elymus dahuricus*	1				1	1				1				1	NE
*Elymus virginicus*		1			0		1			0				1	NE
*Festuca arizonica*	2	2			2				4	NE				4	NE
*Festuca arundinacea*	1	4	1	10	0	1	3	1	11	0	2			14	2
*Festuca eskia*				1	NE				1	NE	1				1
*Festuca pratensis*	1	1			1		1		1	0				2	NE
*Festuca rubra*		2		2	0		1		3	0				4	NE
*Festuca sinensis*	1				1			1		−1				1	NE
*Hordelymus europaeus*		1			0	1				1				1	NE
*Leymus chinensis*	1				1				1	NE				1	NE
*Lolium perenne*	3	4	5	5	−2	2	1	3	9	−1	1			16	1
*Poa alsodes*	1				1				1	NE				1	NE
*Poa autumnalis*	1				1				1	NE				1	NE
**Global effect**					9					1					5

*The arithmetic sum represents the prevailing endophyte-mediated effect on each plant species performance. The variables not evaluated within the drought experiments are abbreviated as NE. Host grass species are in alphabetical order and domesticated species are underlined*.

### Growth and Morphometric Variables

Overall, the symbiosis with endophytes was associated with higher above- and belowground biomass under drought conditions, suggesting an improvement of plant fitness (aboveground biomass: zval = 3.234, *P* = 0.0012; belowground biomass: zval = 2.471, *P* = 0.013; Figure 1). This pattern was replicated only in wild grasses (aboveground biomass_wild species_: zval = 2.719, *P* = 0.006; belowground biomass_wild species_: zval = 2.093, *P* = 0.036) but non-significant on domesticated grasses (aboveground biomass_domesticated species_: zval = 1.325, *P* = 0.185; belowground biomass_domesticated species_: zval = 0.360, *P* = 0.718) either for aboveground or belowground biomass. As a whole, the number of tillers was not different between endophyte-infected and non-infected plants under drought conditions (tillering: zval = 1.421, *P* = 0.155). Nonetheless, the endophyte-mediated promotion of tillering was significant on wild grasses (tillering_wild species_: zval = 2.864, *P* = 0.004). There are two points here worth remarking: (i) the very low number of studies in domesticated grasses (see Supplementary Table 1) and (ii) the high variability in the results among individual studies with wild grasses. It is worth noting that despite studies with domesticated grasses are more numerous in comparison with those on wild grass spp., few of them met the requirements to be included in the quantitative calculations.

### Plant Physiological Variables

Regarding the adaptation mechanisms associated with the “avoidance strategy” (which allow plants to maintain an efficient water balance during drought stress), endophyte-infected plants seemed to develop a more extensive root system (5 out of 12; see also Figure 1), a more efficient stomata regulation (5 out of 7), and a higher accumulation of solutes (4 out of 6) compared to non-infected plants (Table 2). Variables associated with drought tolerance (e.g., those that enable plants to survive a period of water-deficit accumulation of secondary metabolites) were commonly increased in endophyte-infected plants [osmotic potential (7 out of 11), osmotic adjustment (3 out of 4), and water use efficiency (2 out of 4)] (Table 2). In three out of four cases (Table 2), an endophyte-associated improvement in plant performance under drought was observed by boosting plant recovery once drought conditions were relieved. However, endophyte-infected and non-infected plants were not different in the biomass accumulated during the recovery period after a drought event (QM = 2.821, *p* = 0.24). Analyzed independently, the effect size of the drought treatment (pre- vs. post-drought event performance) were on average −1.157 ± 0.69 for E+ [estimate (±SE), zval = −1.67, *P* = 0.09, *n* = 5] and −1.146 ± 0.689 for E– plants [estimate (±SE), zval = −1.661 *P* = 0.096, *n* = 5].

**Table 2:** Summary table showing adaptation mechanisms for the different strategies plant may use to withstand drought conditions (*sensu* Malinowski and Belesky, 2019) and that can be modulated by *Epichloë* fungal endophytes.

**Strategy**	**Adaptation mechanism**	**Observations**	**Sum**	
			**For mechanism**	**For strategy**
Avoidance	Extensive root system	12	5	=16
	Stomata regulation	8	6	
	Accumulation of solutes	8	5	
Tolerance	Accumulation of metabolites	11	7	=15
	Osmotic adjustment	6	5	
	Cell wall elasticity	0	0	
	Water use efficiency	5	3	
Recovery	Higher regrowth	5	4	=4

*By means of assigning +1, 0, and −1 for reported beneficial, neutral, or detrimental effects of endophytes on a given plant adaptation mechanism, the arithmetic sum represents the prevailing endophyte-mediated mechanisms and strategies*.

### Plant Survival

Of the six experiments evaluating endophyte effects on host plant survival during drought (Supplementary Material), only one had merit for being included in the calculus of effect size. In this article, the endophyte-mediated effect in *L. perenne* plant survival was conditional on the origin of populations; it was apparent on plants belonging to populations collected in xeric environments (Gibert et al., 2012). When considering all the studies [*Festuca eskia* (Gibert and Hazard, 2011) and *Bromus laevipes* (Afkhami et al., 2014)], the symbiosis with fungal endophytes improved survival in plant populations facing drought (Table 1).

## Discussion

The idea that *Epichloë* fungal endophytes improve plant tolerance to drought is widespread throughout the literature and is generally mentioned even in papers not dealing with this particular aspect. Our review also shows that there is a strong bias toward the study of this topic in domesticated species—a pattern that had been previously identified in the study of other endophyte-mediated effects on plants (e.g., resistance to herbivores; Saikkonen et al., 2006). In the case of drought, however, the disparity in the number of studies between domesticated and wild species is finally balanced when we considered only those papers with sufficient data quality for quantitative analysis. This may reflect the historical trajectory of the investigation around the endophyte effects on plant tolerance to drought since the early papers using tall fescue or perennial ryegrass as study models did not, usually, provide the information to calculate effect sizes in the meta-analysis (criterion iii; section Materials and Methods). Interestingly, when only papers that met criterion iii are considered, the endophyte effects on variables characterizing plant performance under drought were not significant in domesticated species but significant and positive in wild species. Therefore, this seems to differ from what was observed for the endophyte-conferred protection against herbivores (Saikkonen et al., 2006, 2010) and may be the result of human-driven selection processes for high primary productivity in agricultural settings vs. the natural selection forces shaping the grass–endophyte symbiosis in production-limited environments.

One of the main research goals across the literature has been to determine the underlying mechanisms of the endophyte-mediated effects on host plant performance under drought (Malinowski and Belesky, 2000, 2019; Hamilton et al., 2012; Dastogeer, 2018; Wang et al., 2020). Accordingly, we found several studies linking physiological and morphological response variables with the performance of endophyte-infected and non-infected plants facing drought conditions (Malinowski and Belesky, 2000; Hamilton et al., 2012; Wang et al., 2020). We took the scheme proposed by Malinowski and Belesky (2019) for the different plant mechanisms (here “strategies”) through which plants can maintain a relatively good performance under drought stress. We found that compared to non-infected plants, endophyte-infected plants tended to have higher root biomass (likely associated with a more extensive root system and higher exploration of soil), a better stomata regulation, and a higher accumulation of solutes. These three physiological mechanisms are associated with improved water uptake, reduced transpiration, and higher water storage (Malinowski and Belesky, 2000, 2019; Acuña-Rodríguez et al., 2020). In the same way, the endophyte-mediated higher accumulation of secondary metabolites would be associated with improved osmotic adjustment and water use efficiency allowing individual plants to maintain growth under water restrictions. Besides free sugars, sugar alcohols, and amino acids (Singh et al., 2011), the fungal-alkaloids have been suggested to play roles as osmotic molecules (Nagabhyru et al., 2013), a characteristic that increases the difference between endophyte-infected and non-infected plants. Despite this effort in linking physiology and morphological traits with plant performance under drought conditions, it is interesting to highlight the scarce connection of these findings with plant ecological fitness. No experiment conducted to evaluate the endophyte effect on plant performance under drought uses plant fecundity or seed production as a response variable of fitness. The consideration of the impact of drought on seed production and the transgenerational consequences for progeny performance becomes especially relevant in species associated with vertically transmitted fungal endophytes, since this not only determines the host fitness but also the microorganism persistence (Gundel et al., 2011, 2017; Cavazos et al., 2018; Donald et al., 2021).

Information resulting from experiments in which soil water availability is manipulated and the relative performance of endophyte-infected and non-infected domesticated grass species is evaluated has been usually used to elaborate ecological hypotheses to understand the expression of the symbiosis in nature (e.g., because fungal endophytes increase plant tolerance to drought, higher frequencies of endophyte-infected individuals are expected to occur in arid extremes of precipitation gradients). However, this has yielded contrasting results (Semmartin et al., 2015; Gundel et al., 2016). A recent paper showed that endophyte infection frequency in a population can be highly dynamic, as variation in the environmental context (accounted for interannual variability in precipitation level) may differentially affect any of the underlying processes that determine the symbiosis prevalence, the differential fitness between endophyte-infected and non-infected plants, and the efficiency with which fungal endophytes are transmitted (Donald et al., 2021). Particularly in arid environments, survey studies exploring the occurrence of endophyte-infected plants have constantly shown a low incidence of endophyte-symbiotic plants (Novas et al., 2007; Gundel et al., 2011; Semmartin et al., 2015). As the cost–effect relation of the endophyte symbiosis may vary along water availability gradients (Sneck et al., 2017; Donald et al., 2021), it turns out difficult to establish predictions about the role of endophytes on plant fitness in arid environments based only on the relative performance of plants with and without endophyte under temporary or occasional situations of water shortage. Although plants present specific adaptations to cope with aridity conditions, limitations to primary productivity imposed by permanent situations of water scarcity can make foliar fungal symbionts energetically expensive to maintain (Semmartin et al., 2015; Gundel et al., 2016). In those cases, symbiont-delivered benefits would not payback the maintenance costs.

Even though tall fescue is the most domesticated species for production purposes by humans, its great variety of ecotypes associated with its environment of origin makes it a unique model system for understanding the role of fungal endophytes in plant adaptation (Hand et al., 2010). In Mediterranean climates, plant survival to high summer temperatures and water shortage is a critical fitness trait, and insights on underlying mechanisms to the endophyte effects on this aspect of performance can come from well-studied forage species. The high frequency of endophyte infection in native accessions of Mediterranean tall fescue (Clement et al., 2001; Piano et al., 2005; Pecetti et al., 2007) suggests an important ecological role, similar to that in continental tall fescue ecotypes. Although endophytes often trigger similar responses in summer-dormant and summer-active tall fescue accessions in terms of alkaloids and other metabolites (e.g., phenolic compounds; Assuero et al., 2002; Hamilton et al., 2012; Qawasmeh et al., 2012; Norton et al., 2016), the symbiosis can clearly contribute to plant fitness including survival of high summer temperatures (Malinowski and Belesky, 2019; Acuña-Rodríguez et al., 2020). Summer-dormant accessions of cool-season perennial grasses, including tall fescue, that evolved in the Mediterranean Basin developed an endogenous mechanism of summer dormancy, which represents drought avoidance strategy (Volaire and Norton, 2006). Although the genetic and biochemical bases of summer dormancy mechanism in cool-season perennial grasses are still not well-understood, it has been shown that stem determinacy is a major component of the summer dormancy mechanism in tall fescue, and it is regulated by TERMINAL FLOWER1 (TFL1-like) genes that are homologs of CENTRORADIALIS (CEN) gene sequences (Missaoui et al., 2017). At the biochemical level, oxidative protection of stem meristems during summer drought has been proposed as another important component of the summer dormancy mechanism in cool-season grasses (Malinowski et al., 2005). Some summer-dormant tall fescue ecotypes from the Mediterranean Basin harbor endophytes (designated FaTG-2 and FaTG-3) that are genetically, biochemically, and morphologically different from *E. coenophiala*, the endophyte commonly found in summer-active (continental) tall fescue ecotypes (Christensen et al., 1993; Clement et al., 2001; Piano et al., 2005).

For a better understanding of the relationship between the prevalence of fungal endophytes and their role in plant adaptation to the environment, we need to integrate individual-based studies with those with population-level approaches. In a very few papers, however, demographic approaches using vital rates as response variables (e.g., survival, plant fecundity, recruitment) were used to evaluate the endophyte effects on plant responses to drought. Regarding plant survival, drought conditions in manipulative experiments are seldom so extreme as to cause the death of plants. Although plant survival is a critical demographic variable in population ecology, it can reflect the probability of individual plants surviving the severe stress in physiological studies (McDowell et al., 2008; Volaire, 2018). Therefore, the survival of individuals may be a key response variable on which drought stress can operate as a selective force in favor of endophyte-infected plants in populations, and it merits more attention. Considering that the level and intensity of water shortage in manipulative experiments, are decided based on expert knowledge but limited information, part of the observed variability may be just the result of inappropriate approaches. Nonetheless, the observed variability among species may be explained by the prevailing and past characteristics of the environment of origin. Thus, short-term experiments can be adequate and provide valuable information for domesticated plant species occurring in agricultural settings but give little information for plant species that have evolved in arid ecosystems. For species adapted to arid and semiarid environments, long-term experiments evaluating vital rates and endophyte-symbiont dynamics over time would be fundamental for unveiling the impact of water scarcity (vs. higher restriction, or water addition) on differential fitness of endophyte-infected and non-infected plants, and endophyte transmission.

The above-mentioned aspects could bring some light to understand the sources of the observed variability in results even within-species of the domesticated grasses. These species are generally widely distributed and comprise different ecotypes with divergent strategies such as those described above for tall fescue. Considering this, the short-term experiments designed for studying the impact of punctual drought events on active summer-dormant plants originally from Mediterranean climates might yield inconclusive results, while the same experiments carried out with continental ecotypes show significant responses. This highlights the importance of designing the experiments taking into account the life-history traits of the ecotypes. We hope this work sparks future research addressing general ecological questions on the role of *Epichloë* fungal endophytes in plant adaptation to different environments but considering the difference between the occurrence of drought events in mesic ecosystems from the existing permanent water shortage of arid lands. Particularly useful would be to combine field survey with manipulative experiments along the species distribution range (e.g., Gibert et al., 2012; Afkhami et al., 2014) but especially covering the very arid extreme (i.e., ≤ 400 mm year^−1^; Gundel et al., 2016). In addition to the challenge of linking physiological mechanisms of plant adaptation (fitness) to the different ecological settings (Volaire, 2018), the evaluation of demographic vital rates, such as survival and fecundity, and vertical transmission efficiency of symbionts would allow to identify the endophyte-mediated plant strategies and to better integrate the ecological scales and levels of organization (individual to population; Gundel et al., 2008; Donald et al., 2021). Besides controlling the endophyte-symbiotic status of plants and water availability, those experiments should also take into account the changes in the genetic structure of symbionts' populations, both of the grass and the fungus species (Keith, 2000; Leinonen et al., 2019), to unveil the role of fungal endophytes in plant adaptation to extremely arid conditions. Under current scenarios of global change, it is especially relevant to see whether the association with fungal endophytes will buffer the effect of water shortage and climatic variability, allowing species to maintain or increase the home-range distribution.

## Data Availability Statement

The original contributions presented in the study are included in the article/Supplementary Material, further inquiries can be directed to the corresponding author/s.

## Author Contributions

FD, LP, and PG designed the methodology, collected the data, and led the writing. All authors conceived the ideas and design the work, analyzed and interpreted the data, contributed to the article, and approved the final version to be published.

## Conflict of Interest

The authors declare that the research was conducted in the absence of any commercial or financial relationships that could be construed as a potential conflict of interest.
