# Supplementary material for: A Systematic Review on the Effects of Epichloë Fungal Endophytes on Drought Tolerance in Cool-Season Grasses
Source: Front Plant Sci. 2021 Mar 24;12:644731. doi: 10.3389/fpls.2021.644731 (PMC8025668; doi:10.3389/fpls.2021.644731)

**Supplementary material: Models and outputs**

**Index**

1. Abovegroun biomass.......................1

2. Belowground biomass......................5

3. Tillering.............................................9

4. Physiological variables.....................13

5. Recovery...........................................17

1. Aboveground biomass

Random-Effects Model (k = 21; tau^2 estimator: REML)

 logLik deviance AIC BIC AICc
 -45.1717 90.3434 94.3434 96.3349 95.0493

 tau^2 (estimated amount of total heterogeneity): 3.9648 (SE = 1.4156)
 tau (square root of estimated tau^2 value): 1.9912
 I^2 (total heterogeneity / total variability): 94.45%
 H^2 (total variability / sampling variability): 18.03

 Test for Heterogeneity:
 Q(df = 20) = 147.4698, p-val < .0001

 Model Results:

 estimate se zval pval ci.lb ci.ub
 1.4996 0.4636 3.2347 0.0012 0.5910 2.4083 **

 ---
 Signif. codes: 0 '***' 0.001 '**' 0.01 '*' 0.05 '.' 0.1 ' ' 1


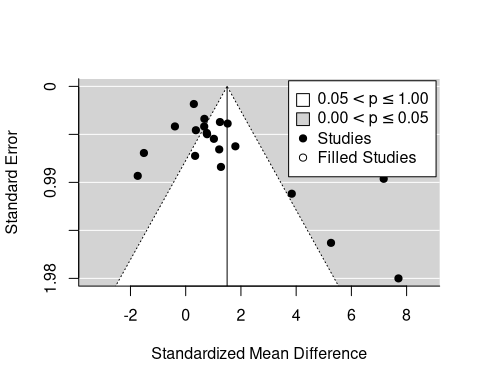


Regression Test for Funnel Plot Asymmetry

 model: weighted regression with multiplicative dispersion
 predictor: standard error

 test for funnel plot asymmetry: t = 2.5035, df = 19, p = 0.0216

Fail-safe N Calculation Using the Rosenthal Approach

 Observed Significance Level: <.0001
 Target Significance Level: 0.05

 Fail-safe N: 734

***# Mixed effects***
*#Mixed effect, factor "wild Vs domesticated"*

Multivariate Meta-Analysis Model (k = 21; method: REML)

 logLik Deviance AIC BIC AICc
 -62.5763 125.1527 131.1527 133.9860 132.7527

 Variance Components:

 estim sqrt nlvls fixed factor
 sigma^2 0.7651 0.8747 16 no id

 Test for Residual Heterogeneity:
 QE(df = 19) = 132.2257, p-val < .0001

 Test of Moderators (coefficients 1:2):
 QM(df = 2) = 9.1505, p-val = 0.0103

 Model Results:

 estimate se zval pval
 factor(wild_domesticated)domesticated 0.5844 0.4410 1.3251 0.1851
 factor(wild_domesticated)wild 0.8773 0.3226 2.7193 0.0065
 ci.lb ci.ub
 factor(wild_domesticated)domesticated -0.2800 1.4488
 factor(wild_domesticated)wild 0.2450 1.5096 **

 ---
 Signif. codes: 0 '***' 0.001 '**' 0.01 '*' 0.05 '.' 0.1 ' ' 1


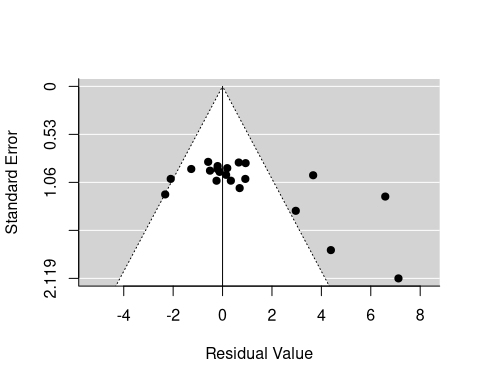


*#Mixed effect, factor "host"*
Multivariate Meta-Analysis Model (k = 21; method: REML)

 logLik Deviance AIC BIC AICc
 -45.7007 91.4014 115.4014 119.0324 427.4014

 Variance Components:

 estim sqrt nlvls fixed factor
 sigma^2 0.4515 0.6719 16 no id

 Test for Residual Heterogeneity:
 QE(df = 10) = 85.8329, p-val < .0001

 Test of Moderators (coefficients 1:11):
 QM(df = 11) = 31.1035, p-val = 0.0011

 Model Results:

 estimate se zval pval ci.lb
 factor(host)Elymus dahuricus 1.7987 0.9129 1.9704 0.0488 0.0095
 factor(host)Elymus virginicus 0.6700 0.7889 0.8494 0.3957 -0.8761
 factor(host)Festuca arizonica 0.2921 0.4726 0.6180 0.5365 -0.6342
 factor(host)Festuca pratensis -0.5435 0.7446 -0.7299 0.4654 -2.0028
 factor(host)Festuca rubra -0.5234 0.9341 -0.5603 0.5753 -2.3542
 factor(host)Festuca sinesis 1.8872 0.8750 2.1569 0.0310 0.1723
 factor(host)Hordelymus europaeus 0.2949 0.6960 0.4238 0.6717 -1.0692
 factor(host)Leymus chinensis 5.2619 1.7481 3.0101 0.0026 1.8357
 factor(host)Lolium perenne 1.5056 0.4830 3.1173 0.0018 0.5590
 factor(host)Poa alsodes 0.6772 0.7506 0.9022 0.3670 -0.7940
 factor(host)Poa autumnalis 0.7598 0.8255 0.9204 0.3574 -0.8582
 ci.ub
 factor(host)Elymus dahuricus 3.5880 *
 factor(host)Elymus virginicus 2.2161
 factor(host)Festuca arizonica 1.2184
 factor(host)Festuca pratensis 0.9158
 factor(host)Festuca rubra 1.3074
 factor(host)Festuca sinesis 3.6021 *
 factor(host)Hordelymus europaeus 1.6591
 factor(host)Leymus chinensis 8.6880 **
 factor(host)Lolium perenne 2.4523 **
 factor(host)Poa alsodes 2.1484
 factor(host)Poa autumnalis 2.3779

 ---
 Signif. codes: 0 '***' 0.001 '**' 0.01 '*' 0.05 '.' 0.1 ' ' 1


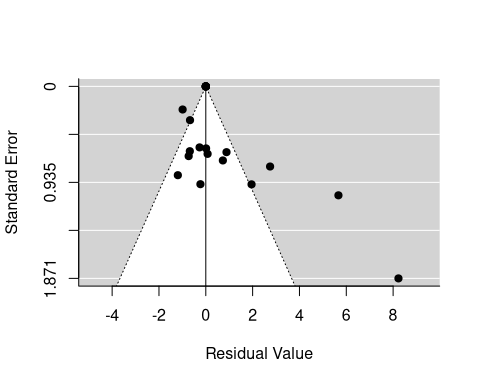


# 2. Belowground biomass

Random-Effects Model (k = 15; tau^2 estimator: REML)

 logLik deviance AIC BIC AICc
 -16.1472 32.2944 36.2944 37.5725 37.3853

 tau^2 (estimated amount of total heterogeneity): 0.1452 (SE = 0.1359)
 tau (square root of estimated tau^2 value): 0.3811
 I^2 (total heterogeneity / total variability): 41.05%
 H^2 (total variability / sampling variability): 1.70

 Test for Heterogeneity:
 Q(df = 14) = 27.2623, p-val = 0.0178

 Model Results:

 estimate se zval pval ci.lb ci.ub
 0.3932 0.1591 2.4719 0.0134 0.0814 0.7049 *

 ---
 Signif. codes: 0 '***' 0.001 '**' 0.01 '*' 0.05 '.' 0.1 ' ' 1

funnel(bg_rmodel)


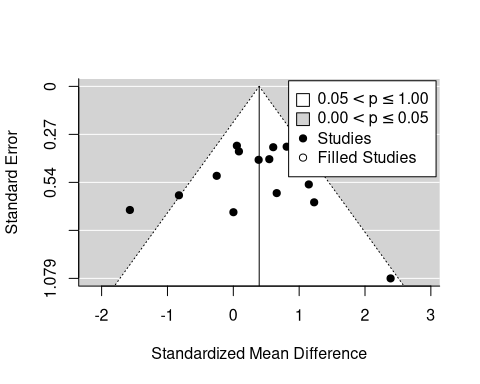


Regression Test for Funnel Plot Asymmetry

 model: weighted regression with multiplicative dispersion
 predictor: standard error

 test for funnel plot asymmetry: t = -0.3050, df = 13, p = 0.7652

Fail-safe N Calculation Using the Rosenthal Approach

 Observed Significance Level: 0.0005
 Target Significance Level: 0.05

 Fail-safe N: 45

***# Mixed effects***
*#Mixed effect, factor "wild Vs domesticated"*

Multivariate Meta-Analysis Model (k = 15; method: REML)

 logLik Deviance AIC BIC AICc
 -14.3170 28.6339 34.6339 36.3288 37.3006

 Variance Components:

 estim sqrt nlvls fixed factor
 sigma^2 0.2385 0.4883 11 no id

 Test for Residual Heterogeneity:
 QE(df = 13) = 26.4185, p-val = 0.0149

 Test of Moderators (coefficients 1:2):
 QM(df = 2) = 4.5144, p-val = 0.1046

 Model Results:

 estimate se zval pval
 factor(wild_domesticated)domesticated 0.1282 0.3559 0.3603 0.7186
 factor(wild_domesticated)wild 0.5136 0.2453 2.0939 0.0363
 ci.lb ci.ub
 factor(wild_domesticated)domesticated -0.5693 0.8258
 factor(wild_domesticated)wild 0.0329 0.9944 *

 ---
 Signif. codes: 0 '***' 0.001 '**' 0.01 '*' 0.05 '.' 0.1 ' ' 1


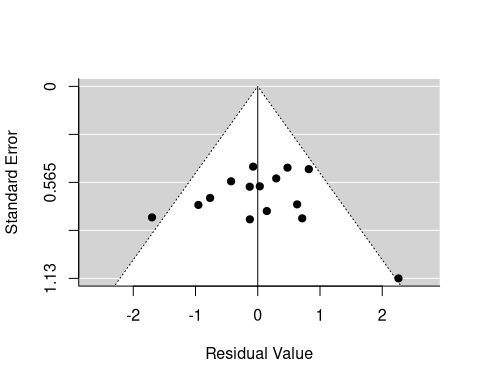


*#Mixed effect, factor "host"*

 Multivariate Meta-Analysis Model (k = 15; method: REML)

 logLik Deviance AIC BIC AICc
 -5.9339 11.8678 31.8678 29.7854 251.8678

 Variance Components:

 estim sqrt nlvls fixed factor
 sigma^2 0.3819 0.6180 11 no id

 Test for Residual Heterogeneity:
 QE(df = 6) = 8.9275, p-val = 0.1777

 Test of Moderators (coefficients 1:9):
 QM(df = 9) = 9.5111, p-val = 0.3915

 Model Results:

 estimate se zval pval ci.lb
 factor(host)Ammophila breviligulata 0.0851 0.7179 0.1186 0.9056 -1.3219
 factor(host)Elymus dahuricus 1.1463 0.8279 1.3846 0.1662 -0.4764
 factor(host)Elymus virginicus 0.5465 0.7411 0.7375 0.4608 -0.9059
 factor(host)Festuca arizonica 0.0884 0.5434 0.1627 0.8708 -0.9766
 factor(host)Festuca pratensis 0.8686 0.7494 1.1591 0.2464 -0.6001
 factor(host)Festuca rubra -1.1512 0.7699 -1.4953 0.1348 -2.6601
 factor(host)Festuca sinesis 0.9200 0.7593 1.2116 0.2257 -0.5682
 factor(host)Lolium perenne 0.5190 0.6489 0.7998 0.4238 -0.7528
 factor(host)Poa alsodes 0.8104 0.7048 1.1499 0.2502 -0.5709
 ci.ub
 factor(host)Ammophila breviligulata 1.4921
 factor(host)Elymus dahuricus 2.7690
 factor(host)Elymus virginicus 1.9990
 factor(host)Festuca arizonica 1.1534
 factor(host)Festuca pratensis 2.3374
 factor(host)Festuca rubra 0.3577
 factor(host)Festuca sinesis 2.4082
 factor(host)Lolium perenne 1.7907
 factor(host)Poa alsodes 2.1918

 ---
 Signif. codes: 0 '***' 0.001 '**' 0.01 '*' 0.05 '.' 0.1 ' ' 1


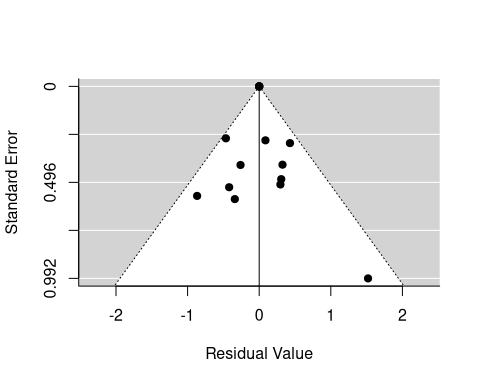


# 3. Tillering

Random-Effects Model (k = 24; tau^2 estimator: REML)

 logLik deviance AIC BIC AICc
 -34.3110 68.6221 72.6221 74.8930 73.2221

 tau^2 (estimated amount of total heterogeneity): 0.5686 (SE = 0.2542)
 tau (square root of estimated tau^2 value): 0.7540
 I^2 (total heterogeneity / total variability): 76.53%
 H^2 (total variability / sampling variability): 4.26

 Test for Heterogeneity:
 Q(df = 23) = 71.5125, p-val < .0001

 Model Results:

 estimate se zval pval ci.lb ci.ub
 0.2722 0.1915 1.4213 0.1552 -0.1032 0.6476

 ---
 Signif. codes: 0 '***' 0.001 '**' 0.01 '*' 0.05 '.' 0.1 ' ' 1


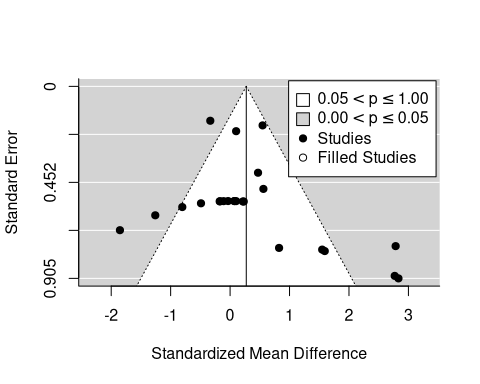


Regression Test for Funnel Plot Asymmetry

 model: weighted regression with multiplicative dispersion
 predictor: standard error

 test for funnel plot asymmetry: t = 1.2955, df = 22, p = 0.2086

Fail-safe N Calculation Using the Rosenthal Approach

 Observed Significance Level: 0.0069
 Target Significance Level: 0.05

 Fail-safe N: 30

***# Mixed effects***
*#Mixed effect, factor "wild Vs domesticated"*

 Multivariate Meta-Analysis Model (k = 24; method: REML)

 logLik Deviance AIC BIC AICc
 -24.6510 49.3020 55.3020 58.5751 56.6353

 Variance Components:

 estim sqrt nlvls fixed factor
 sigma^2 0.7700 0.8775 10 no id

 Test for Residual Heterogeneity:
 QE(df = 22) = 50.4331, p-val = 0.0005

 Test of Moderators (coefficients 1:2):
 QM(df = 2) = 8.3966, p-val = 0.0150

 Model Results:

 estimate se zval pval
 factor(wild_domesticated)domesticated 0.2085 0.4738 0.4399 0.6600
 factor(wild_domesticated)wild 1.1821 0.4127 2.8641 0.0042
 ci.lb ci.ub
 factor(wild_domesticated)domesticated -0.7202 1.1372
 factor(wild_domesticated)wild 0.3732 1.9910 **

 ---
 Signif. codes: 0 '***' 0.001 '**' 0.01 '*' 0.05 '.' 0.1 ' ' 1


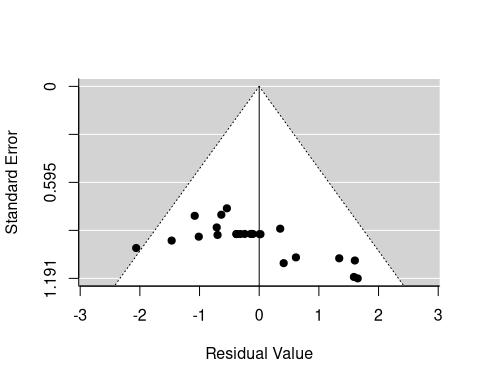


*#Mixed effect, factor "host"*

Multivariate Meta-Analysis Model (k = 24; method: REML)

 logLik Deviance AIC BIC AICc
 -13.0411 26.0822 44.0822 51.0355 74.0822

 Variance Components:

 estim sqrt nlvls fixed factor
 sigma^2 0.0000 0.0000 10 no id

 Test for Residual Heterogeneity:
 QE(df = 16) = 15.9515, p-val = 0.4564

 Test of Moderators (coefficients 1:8):
 QM(df = 8) = 57.8658, p-val < .0001

 Model Results:

 estimate se zval pval
 factor(host)Elymus dahuricus 2.7851 0.7525 3.7013 0.0002
 factor(host)Elymus virginicus 0.4702 0.4068 1.1559 0.2477
 factor(host)Festuca rubra 0.1014 0.2110 0.4809 0.6306
 factor(host)Festuca sinesis 2.8006 0.6354 4.4078 <.0001
 factor(host)Hordelymus europaeus 0.5472 0.1837 2.9784 0.0029
 factor(host)Leymus chinensis 1.5924 0.7758 2.0526 0.0401
 factor(host)Lolium perenne -0.2505 0.1088 -2.3025 0.0213
 factor(host)Schedonorus arundinaceus 1.1831 0.5409 2.1874 0.0287
 ci.lb ci.ub
 factor(host)Elymus dahuricus 1.3103 4.2599 ***
 factor(host)Elymus virginicus -0.3271 1.2674
 factor(host)Festuca rubra -0.3120 0.5149
 factor(host)Festuca sinesis 1.5553 4.0460 ***
 factor(host)Hordelymus europaeus 0.1871 0.9073 **
 factor(host)Leymus chinensis 0.0718 3.1129 *
 factor(host)Lolium perenne -0.4637 -0.0373 *
 factor(host)Schedonorus arundinaceus 0.1230 2.2432 *

 ---
 Signif. codes: 0 '***' 0.001 '**' 0.01 '*' 0.05 '.' 0.1 ' ' 1


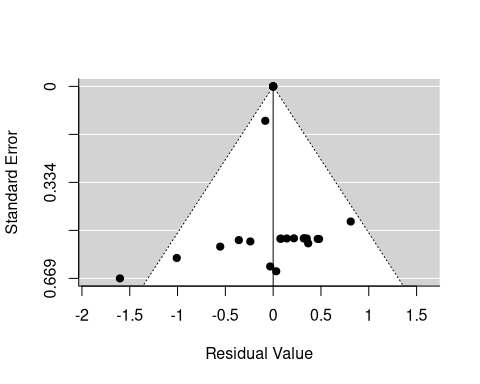


# 4. Physiological variables

Random-Effects Model (k = 20; tau^2 estimator: REML)

 logLik deviance AIC BIC AICc
 -42.1714 84.3428 88.3428 90.2317 89.0928

 tau^2 (estimated amount of total heterogeneity): 3.6216 (SE = 1.3443)
 tau (square root of estimated tau^2 value): 1.9031
 I^2 (total heterogeneity / total variability): 91.97%
 H^2 (total variability / sampling variability): 12.45

 Test for Heterogeneity:
 Q(df = 19) = 153.0468, p-val < .0001

 Model Results:

 estimate se zval pval ci.lb ci.ub
 1.6462 0.4576 3.5972 0.0003 0.7493 2.5431 ***

 ---
 Signif. codes: 0 '***' 0.001 '**' 0.01 '*' 0.05 '.' 0.1 ' ' 1


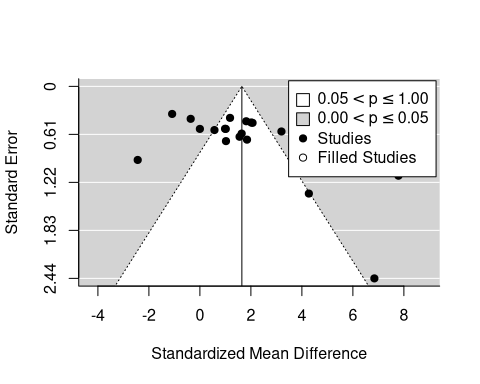


Regression Test for Funnel Plot Asymmetry

 model: weighted regression with multiplicative dispersion
 predictor: standard error

 test for funnel plot asymmetry: t = 2.6307, df = 18, p = 0.0170

Fail-safe N Calculation Using the Rosenthal Approach

 Observed Significance Level: <.0001
 Target Significance Level: 0.05

 Fail-safe N: 763

***# Mixed effects***
*#Mixed effect, factor "wild Vs domesticated"*

 Multivariate Meta-Analysis Model (k = 20; method: REML)

 logLik Deviance AIC BIC AICc
 -52.1420 104.2841 110.2841 112.9552 111.9983

 Variance Components:

 estim sqrt nlvls fixed factor
 sigma^2 1.8312 1.3532 8 no id

 Test for Residual Heterogeneity:
 QE(df = 18) = 152.8387, p-val < .0001

 Test of Moderators (coefficients 1:2):
 QM(df = 2) = 3.3195, p-val = 0.1902

 Model Results:

 estimate se zval pval
 factor(wild_domesticated)domesticated 1.6347 0.9616 1.7000 0.0891
 factor(wild_domesticated)wild 0.4164 0.6353 0.6554 0.5122
 ci.lb ci.ub
 factor(wild_domesticated)domesticated -0.2500 3.5194 .
 factor(wild_domesticated)wild -0.8288 1.6615

 ---
 Signif. codes: 0 '***' 0.001 '**' 0.01 '*' 0.05 '.' 0.1 ' ' 1


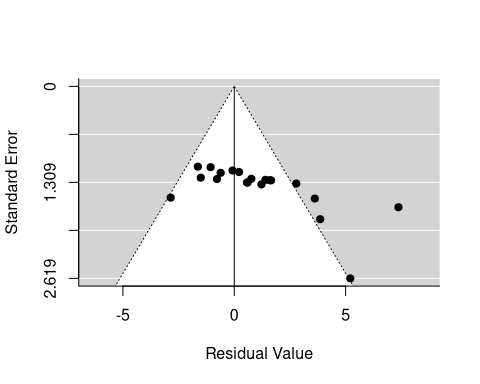


*#Mixed effect, factor "host"*

 Multivariate Meta-Analysis Model (k = 20; method: REML)

 logLik Deviance AIC BIC AICc
 -41.5474 83.0947 97.0947 101.5681 115.7614

 Variance Components:

 estim sqrt nlvls fixed factor
 sigma^2 0.7817 0.8841 8 no id

 Test for Residual Heterogeneity:
 QE(df = 14) = 78.7956, p-val < .0001

 Test of Moderators (coefficients 1:6):
 QM(df = 6) = 16.1239, p-val = 0.0131

 Model Results:

 estimate se zval pval
 factor(host)Achnaterum inebrians 2.0996 0.9068 2.3153 0.0206
 factor(host)Festuca arizonica 0.5958 0.6751 0.8825 0.3775
 factor(host)Leymus chinensis -0.2890 1.1728 -0.2465 0.8053
 factor(host)Lolium perenne 6.8431 2.5949 2.6371 0.0084
 factor(host)Poa alsodes -1.0883 0.9509 -1.1445 0.2524
 factor(host)Schedonorus arundinaceus 0.9249 0.7180 1.2882 0.1977
 ci.lb ci.ub
 factor(host)Achnaterum inebrians 0.3223 3.8770 *
 factor(host)Festuca arizonica -0.7274 1.9189
 factor(host)Leymus chinensis -2.5876 2.0095
 factor(host)Lolium perenne 1.7571 11.9290 **
 factor(host)Poa alsodes -2.9521 0.7754
 factor(host)Schedonorus arundinaceus -0.4823 2.3322

 ---
 Signif. codes: 0 '***' 0.001 '**' 0.01 '*' 0.05 '.' 0.1 ' ' 1


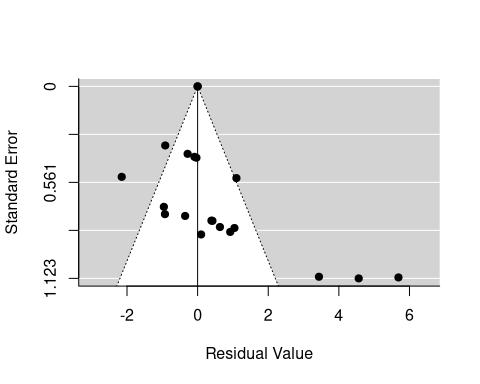


*#Mixed effect, factor "parameter"*
 Multivariate Meta-Analysis Model (k = 20; method: REML)

 logLik Deviance AIC BIC AICc
 -46.7158 93.4315 101.4315 104.7644 104.7649

 Variance Components:

 estim sqrt nlvls fixed factor
 sigma^2 2.0051 1.4160 8 no id

 Test for Residual Heterogeneity:
 QE(df = 17) = 149.0986, p-val < .0001

 Test of Moderators (coefficients 1:3):
 QM(df = 3) = 14.3665, p-val = 0.0024

 Model Results:

 estimate se zval pval ci.lb ci.ub
 factor(param)fot 1.5255 0.5951 2.5634 0.0104 0.3591 2.6918 *
 factor(param)iaf 1.2108 0.9274 1.3055 0.1917 -0.6070 3.0285
 factor(param)trans 0.2472 0.5784 0.4273 0.6691 -0.8865 1.3808

 ---
 Signif. codes: 0 '***' 0.001 '**' 0.01 '*' 0.05 '.' 0.1 ' ' 1


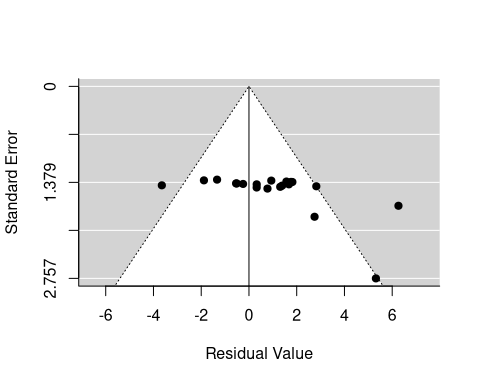


# 5. Recovery

Random-Effects Model (k = 9; tau^2 estimator: REML)

 logLik deviance AIC BIC AICc
 -14.1614 28.3227 32.3227 32.4816 34.7227

 tau^2 (estimated amount of total heterogeneity): 1.4906 (SE = 0.8560)
 tau (square root of estimated tau^2 value): 1.2209
 I^2 (total heterogeneity / total variability): 96.26%
 H^2 (total variability / sampling variability): 26.76

 Test for Heterogeneity:
 Q(df = 8) = 191.8577, p-val < .0001

 Model Results:

 estimate se zval pval ci.lb ci.ub
 -0.7027 0.4403 -1.5957 0.1105 -1.5657 0.1604

 ---
 Signif. codes: 0 '***' 0.001 '**' 0.01 '*' 0.05 '.' 0.1 ' ' 1


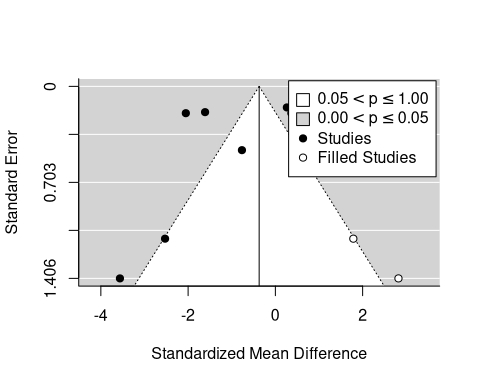

 Regression Test for Funnel Plot Asymmetry

 model: weighted regression with multiplicative dispersion
 predictor: standard error

 test for funnel plot asymmetry: t = -0.5269, df = 7, p = 0.6145

Fail-safe N Calculation Using the Rosenthal Approach

 Observed Significance Level: <.0001
 Target Significance Level: 0.05

 Fail-safe N: 96

***# Mixed effects***
*#Mixed effect, factor "wild Vs domesticated"*

 Multivariate Meta-Analysis Model (k = 9; method: REML)

 logLik Deviance AIC BIC AICc
 -4.2113 8.4226 14.4226 14.2603 22.4226

 Variance Components:

 estim sqrt nlvls fixed factor
 sigma^2 0.5341 0.7308 4 no id

 Test for Residual Heterogeneity:
 QE(df = 7) = 11.6144, p-val = 0.1140

 Test of Moderators (coefficients 1:2):
 QM(df = 2) = 11.3464, p-val = 0.0034

 Model Results:

 estimate se zval pval ci.lb
 factor(wild_dom)domesticated -1.6813 0.5057 -3.3243 0.0009 -2.6725
 factor(wild_dom)wild 0.4006 0.7372 0.5434 0.5868 -1.0443
 ci.ub
 factor(wild_dom)domesticated -0.6900 ***
 factor(wild_dom)wild 1.8456

 ---
 Signif. codes: 0 '***' 0.001 '**' 0.01 '*' 0.05 '.' 0.1 ' ' 1


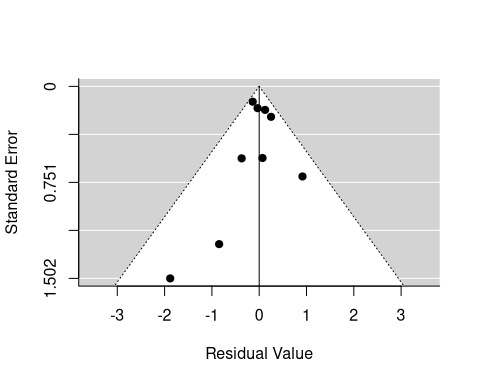


*#Mixed effect, factor "endophyte status"*

 Multivariate Meta-Analysis Model (k = 9; method: REML)

 logLik Deviance AIC BIC AICc
 -7.6869 15.3737 21.3737 21.2115 29.3737

 Variance Components:

 estim sqrt nlvls fixed factor
 sigma^2 1.6730 1.2935 4 no id

 Test for Residual Heterogeneity:
 QE(df = 7) = 189.2741, p-val < .0001

 Test of Moderators (coefficients 1:2):
 QM(df = 2) = 2.8214, p-val = 0.2440

 Model Results:

 estimate se zval pval ci.lb ci.ub
 factor(endo)negative -1.1460 0.6897 -1.6616 0.0966 -2.4979 0.2058 .
 factor(endo)positive -1.1578 0.6919 -1.6734 0.0942 -2.5139 0.1983 .

 ---
 Signif. codes: 0 '***' 0.001 '**' 0.01 '*' 0.05 '.' 0.1 ' ' 1


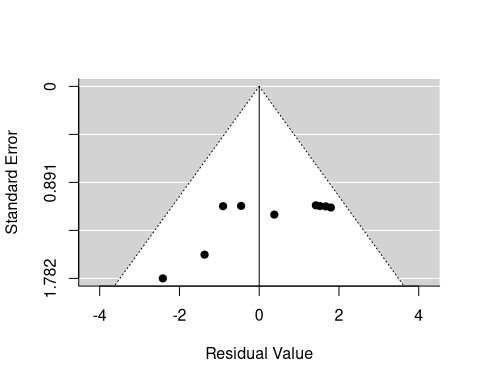

Supplement: Supplementary file 1 [file Data_Sheet_1.docx]
