# Supplementary material for: A Systematic Review on the Effects of Epichloë Fungal Endophytes on Drought Tolerance in Cool-Season Grasses
Source: Front Plant Sci. 2021 Mar 24;12:644731. doi: 10.3389/fpls.2021.644731 (PMC8025668; doi:10.3389/fpls.2021.644731)
Supplement: Supplementary file 2 [file Data_Sheet_2.doc]

**Figure S1. PRISMA flow diagram. The number of records screened is equal to the sum of the number of records initially identified in Scopus after removing duplicated studies and incorporating records identified through reference lists of After exclusions of non-subject articles during the screening phase, 83 full-text text articles were analyzed, 57 of which were excluded leaving a total of 26 articles that were included in the Meta Analysis**

**Screening**

**Included**

**Eligibility**

**Identification**

Records identified through database searching

Scopus records identified (n = 188)
(n =188)

Additional records identified through reference lists of selected articles (n = 2)

Records after duplicates removed
(n = 190)

Records screened
(n = 190)

Records excluded
(n = 107)

- Other endophytes
- Other stresses

Full-text articles assessed for eligibility
(n = 83)

Full-text articles excluded, with reasons
(n = 57)

- Reviews (n = 19)
- Case reports (n = 1)
- Insufficient data to calculate effect size (n = 37)

Studies included in qualitative synthesis
(n = 26)

Studies included in quantitative synthesis (meta-analysis)
(n = 26)
